# A Case of Dropped Head Syndrome Improving in the Early Postoperative Period After Surgery for a Partially Thrombosed Vertebral Artery Aneurysm

**DOI:** 10.1155/crnm/1390710

**Published:** 2026-03-01

**Authors:** Shinnosuke Hashida, Tomoya Kamide, Sho Takata, Daisuke Wajima, Kouichi Misaki, Mitsutoshi Nakada

**Affiliations:** ^1^ Department of Neurosurgery, Kanazawa University, Kanazawa, Ishikawa, Japan, kanazawa-u.ac.jp

**Keywords:** dropped head syndrome, partially thrombosed aneurysm, transcondylar fossa approach, vertebral artery aneurysm

## Abstract

**Introduction:**

Dropped head syndrome (DHS) is associated with various diseases and syndromes, and its cause is sometimes difficult to identify. Partially thrombosed or thrombosed aneurysms can cause neurological symptoms owing to the mass effect and the compression of the surrounding structures. However, no DHS cases due to the mass effect of thrombosed aneurysms have been reported. Herein, we report a case of DHS, which improved after surgery for a partially thrombosed vertebral artery (VA) aneurysm.

**Case Presentation:**

A female patient in her fifties, with a medical history of subarachnoid hemorrhage and consistent partial paralysis of the left leg, experienced a gradual downward shift of gaze while walking. She was diagnosed with suspected DHS, and a large partially thrombosed aneurysm of the left VA was incidentally detected. The disorder in the right lower extremity worsened rapidly, and a mass effect of the aneurysm was suspected. Within 2 days postoperatively, she was able to walk independently. In the early postoperative period (postoperative day 2), cervical extensor weakness improved, allowing maintenance of a horizontal gaze.

**Discussion:**

This is the first report describing an association between DHS and a large partially thrombosed VA aneurysm. DHS improved in the early postoperative period following surgery. Although the exact mechanism remains unclear, this case suggests that factors beyond immediate radiographic decompression may be involved.

## 1. Introduction

Dropped head syndrome (DHS) is a severe condition characterized by the inability to maintain an upright position of the neck and is often associated with various diseases or syndromes [1–6]. The conditions linked to DHS are typically categorized into neurological, neuromuscular, muscular, and other etiologies, making it challenging to determine the precise cause of DHS in some cases. Although large or giant partially thrombosed aneurysms typically present with symptoms related to the mass effect and the compression of the surrounding structures [7], there are no known reports of DHS caused by aneurysms with intraluminal thrombosis. We report the case of a patient with DHS who showed improvement after surgery for a large partially thrombosed vertebral artery (VA) aneurysm.

## 2. Case Presentation

**FIGURE 1:**
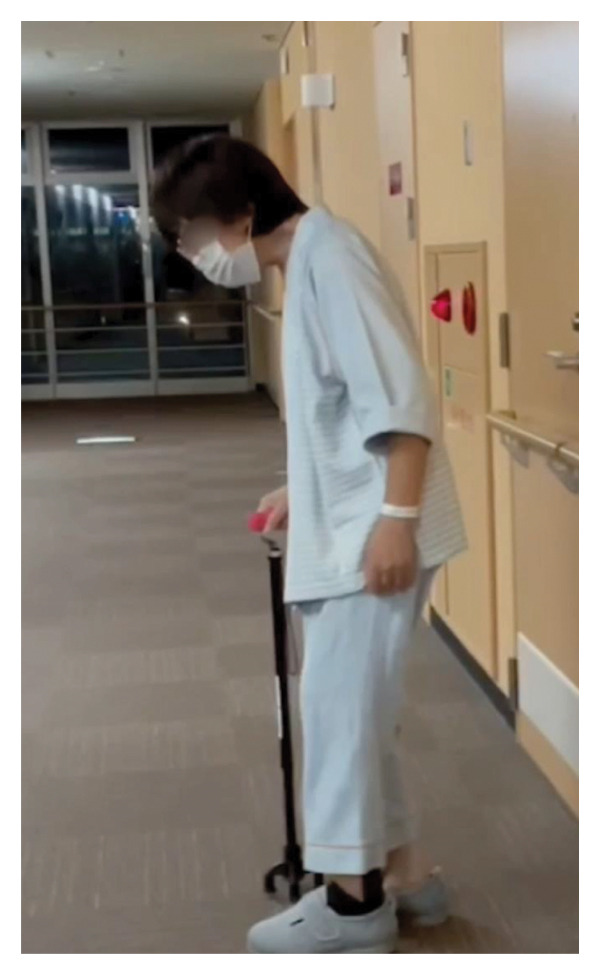
The patient is unable to keep a horizontal gaze and keep looking down.

**FIGURE 2:**
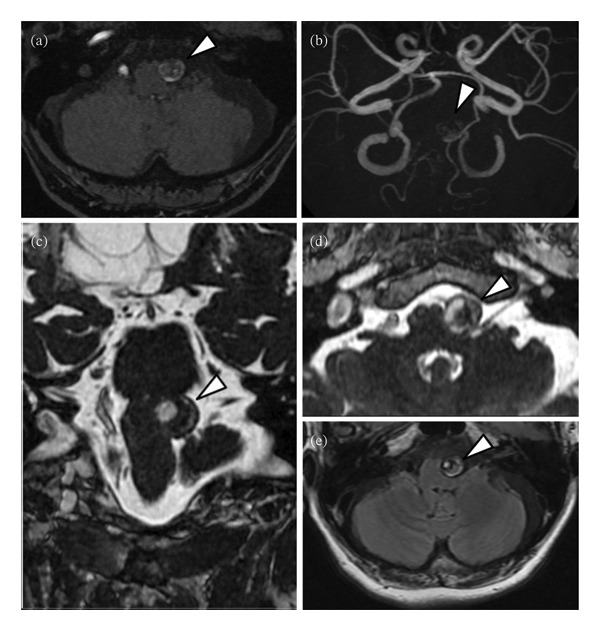
Preoperative magnetic resonance imaging (MRI). Magnetic resonance angiography (MRA) time‐of‐flight image (axial (a); maximum intensity projection (b) shows a large left vertebral artery partially thrombosed aneurysm (13.2 × 13.5 mm). Gadolinium (Gd)−enhanced fast imaging employing steady state acquisition (FIESTA) (coronal (c); axial (d)) shows the aneurysm compressing the brain stem from left to right. Fluid attenuated inversion recovery (FLAIR) (e) shows no brain stem edema due to the mass effect of the aneurysm. Arrowhead: partially thrombosed aneurysm.

**FIGURE 3:**
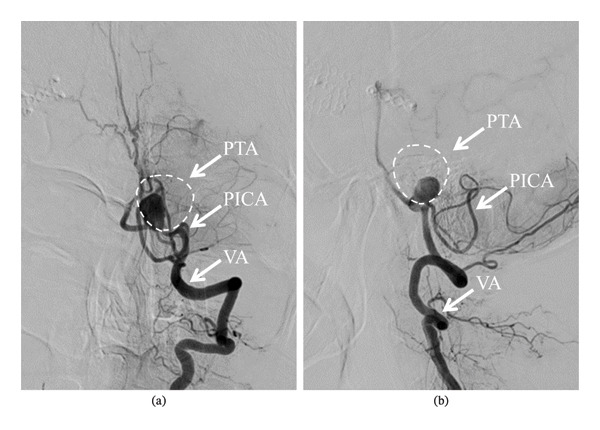
Left vertebral angiography performed before surgery (anteroposterior (a); lateral (b)) shows residual blood flow into the partially thrombosed aneurysm, suggesting that posterior inferior cerebellar artery (PICA) originates from the aneurysm dome. The dotted circle indicates the estimated outer diameter of the partially thrombosed aneurysm based on MRI measurements; PICA: posterior inferior cerebellar artery, PTA: partially thrombosed aneurysm, VA: vertebral artery.

**FIGURE 4:**
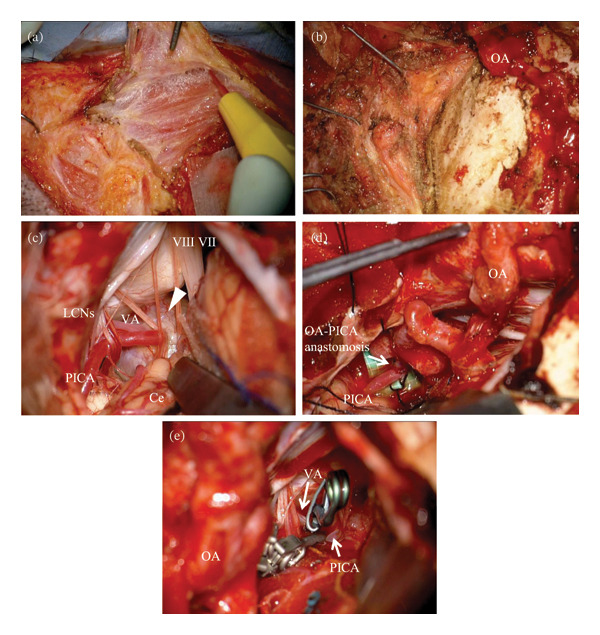
Intraoperative findings. Suboccipital muscles are detached layer‐by‐layer and retracted, respectively, with dissection of OA from the surrounding tissue to perform OA−PICA anastomosis. (a, b) VA and PICA originated from the aneurysm’s dome, and distal VA behind the aneurysm is not confirmed. (c) As planned before procedure, OA−PICA anastomosis is performed. (d) After checking blood flow via the anastomosis, clips are applied to the proximal VA and PICA. (e) Arrowhead: partially thrombosed aneurysm, VII: facial nerve, VIII: vestibular nerve, Ce: cerebellum, LCNs: lower cranial nerves, OA: occipital artery, PICA: posterior inferior cerebellar artery, VA: vertebral artery.

**FIGURE 5:**
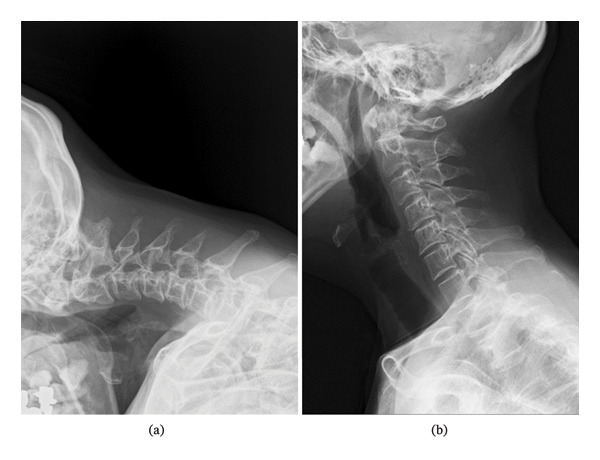
Lateral cervical radiographs. Before the surgery for the aneurysm, chin‐to‐chest phenomenon is observed, indicating dropped head syndrome (a). Dramatically improved cervical extension right after the surgery (b).

A female patient in her fifties was introduced to our department. She had experienced partial paralysis of the left leg for 20 years, following a subarachnoid hemorrhage caused by a ruptured anterior communicating artery aneurysm and had been walking with the aid of a leg orthosis. Over the past year, she had found it increasingly difficult to maintain a straight head while walking (Figure 1), and thus sought consultation with the orthopedic and neurological departments. She could not extend her neck because of cervical muscle weakness, leading to the suspicion of DHS. Although numerous examinations were conducted, the specific etiology of the syndrome could not be identified. Magnetic resonance imaging (MRI) incidentally revealed a large left VA−partially thrombosed aneurysm measuring 13. × 13.5 mm, compressing the brainstem from left to right; however, there was no fluid‐attenuated inversion recovery hyperintensity in the brainstem, indicative of edema due to the mass effect (Figure 2). Consequently, the aneurysm was deemed asymptomatic, and observation and conservative treatment were recommended. Subsequently, the patient experienced a rapid worsening of her right lower extremity function and was admitted through the emergency department. Although there was still no evidence of brainstem edema on MRI, we considered the aneurysm to be the cause of the patient’s gait disturbance due to its mass effect. Digital subtraction angiography demonstrated that the VA and posterior inferior cerebellar artery (PICA) originated from the aneurysm’s dome (Figure 3). Trapping the aneurysm with an occipital artery (OA)−PICA anastomosis was planned following thrombectomy to reduce the mass effect. We proceeded with the transcondylar fossa approach using the park‐bench position on the right side, and a C‐shaped skin incision was made from the inion to the mastoid tip. The occipital muscles were incised, detached, and reflected as a single layer, with the OA harvesting. Lateral suboccipital craniectomy was performed, exposing the aneurysm along the VA and confirming that the PICA originated from the dome of the aneurysm. As we were unable to access the distal VA, we performed an OA−PICA anastomosis and only proximal clipping of the VA and PICA without thrombectomy. The muscle layers were approximated and attached to the original positions layer by layer, and the procedure was completed (Figure 4). The postoperative course was uneventful, and 2 days after the surgery, her right leg weakness improved, and unexpectedly, her DHS also improved. MRI performed 1 week postoperatively showed a slight reduction in brainstem compression caused by the aneurysm. One year after the surgery, the patient remained ambulatory with a maintained horizontal gaze, and there was no recurrence of right lower extremity dysfunction or DHS (Figure 5).

## 3. Discussion

DHS is a clinical condition characterized by weakness of the neck extensor muscles, with more than two‐thirds of all patients classified into just four primary diagnostic categories: isolated neck extensor myopathy (31.8%), Parkinson’s disease (20.2%), myasthenia gravis (12.4%), and amyotrophic lateral sclerosis (7.9%) [1–6]. Although many causes have been reported, to the best of our knowledge, there are no reports documenting a relationship between DHS and partially thrombosed aneurysms. The choice of treatment depends on the underlying etiology of DHS and may include corticosteroid, intravenous immunoglobulin, L‐dopa, and thyroxine replacement [4, 5, 8, 9]. The overall positive response rate to treatment is 64.3%, with primary medical treatment, immune suppression, and a combination of both demonstrating success rates of 73.5%, 78.9%, and 87.5%, respectively [2, 10]. Some studies have indicated that conservative measures such as observation, bracing, and physical therapy yield little to no benefit, or only 18.2%–20.9% of patients experience recovery [1, 11–13]. Conversely, one report demonstrated that 70−80% of patients with DHS improved following a novel rehabilitation program over a 2‐week period [14]. Notably, patients with acute‐onset DHS (within 3 months) or those who received early first intervention (average: 3.6 months) have been reported to have better outcomes with conservative treatment compared to patients with chronic‐onset DHS (over 3 months), or who received delayed first interventions during 4− and 24 months [12, 15]. Therefore, it is essential to investigate which physical therapy methods are most effective in relieving DHS.

If nonsurgical interventions prove ineffective or the etiology remains unidentified, surgical options should be carefully considered [10]. The goals of surgical treatment of DHS are to improve neck extensor function, correct spine alignment, and decompress neural structures. Surgical intervention, specifically cervicothoracic arthrodesis, has been reported to improve horizontal gaze in 91.3% of patients, and overall surgery has been reported to be successful in 93.8% of patients in another systematic review [2, 10]. In contrast, the failure rate for patients undergoing cervical arthrodesis alone without thoracic extension has been reported to be 71% [10]. Thus, extending the fusion to the upper thoracic segments between T1 and T5 may provide more stable biomechanical support to the cervical construct and reduce the risk of failure [1, 10]. In addition, several surgical techniques, including anterior release, posterior fusion, and laminectomy, have been documented [10, 16]. However, to the best of our knowledge, there are no reports on the surgical reconstruction of cervical muscles alone.

Thrombosed aneurysms cause neurological symptoms owing to the mass effect on the brainstem, often accompanied by edema on MRI [7, 17]. The mass effect on the brainstem can lead to neurological deficits, indicating that the aneurysm is symptomatic and requires further intervention. Treatment typically involves techniques beyond conventional clipping, such as thrombectomy with clip reconstruction or bypass with parent artery occlusion [17]. Occlusion of the vasa vasorum is crucial because incomplete clipping or coiling without parent vessel occlusion can lead to suboptimal outcomes [17].

In our case, we hypothesized that the patient’s right leg walking disorder was due to aneurysmal compression of the pyramidal tract. Additionally, given the lesion’s proximity to the jugular foramen, lower cranial nerve involvement was considered; however, there were no clinical signs of IX–XI palsy and no imaging evidence of direct nerve compression, and thus this mechanism was considered unlikely [18, 19].

The early postoperative improvement in DHS occurred without substantial radiographic reduction of brainstem compression, suggesting that mechanisms other than immediate decompression may have contributed. During the transcondylar fossa approach, suboccipital muscles were detached and subsequently reapproximated to their original positions, rather than reconstructed. Therefore, we interpret this finding as hypothesis‐generating, and alternative explanations—such as postoperative pain relief, changes in compensatory posture, restoration of muscle tension/length after reapproximation, or early rehabilitation effects—should be considered.

## 4. Conclusion

This is the first report to demonstrate a possible connection between DHS and mass effect of a partially thrombosed aneurysm. DHS improved in the early postoperative period after surgery. The mechanism remains uncertain; in addition to a potential mass effect, perioperative factors including muscle detachment and reapproximation and postoperative functional changes may have contributed.

## Funding

The authors have not received funding from any sources.

## Disclosure

This manuscript is original and has not been submitted elsewhere in part or entirety.

## Consent

The patient has consented to the submission of the case report to the journal.

## Conflicts of Interest

The authors declare no conflicts of interest.

## Data Availability

The data that support the findings of this study are available upon request from the corresponding author. The data is not publicly available due to privacy or ethical restrictions.
